# Decoding episodic memory in ageing: A Bayesian analysis of activity patterns predicting memory

**DOI:** 10.1016/j.neuroimage.2011.08.071

**Published:** 2012-01-16

**Authors:** Alexa M. Morcom, Karl J. Friston

**Affiliations:** a Psychology and Centre for Cognitive Ageing and Cognitive Epidemiology, University of Edinburgh, 7 George Square, Edinburgh EH8 9JZ, UK; b Centre for Cognitive and Neural Systems, University of Edinburgh, 1 George Square, Edinburgh EH8 9JZ, UK; c The Wellcome Trust Centre for Neuroimaging, Institute of Neurology, University College London, 12 Queen Square, London, WC1N 3BG, UK

**Keywords:** Ageing, fMRI, Episodic memory, Multivariate, Decoding, Over-recruitment, Dedifferentiation, Compensation, Prefrontal cortex, Lateralisation

## Abstract

Normal ageing is associated with a decline in episodic memory, and neuroimaging studies in older adults have shown reduced activity in prefrontal cortex and other regions critical for memory function in the young. However, older adults also activate additional regions, suggesting a degree of functional reorganisation that has been attributed variously to detrimental and adaptive changes. Evaluation of these competing hypotheses depends critically upon inferences about the relative location and distribution of activity that are not well supported by current univariate or multivariate analyses. Here, we employed a recently developed model-based multivariate ‘decoding’ approach (Friston et al., 2008) to re-analyse a rich episodic encoding dataset and examine directly how the patterns of activity change in ageing. We assessed which spatial activity patterns, within lateral prefrontal cortex, best predict successful memory formation. Bayesian model comparison showed that the older adults had more distributed and bilateral (fragmented) predictive patterns of activity in anterior inferior frontal gyrus and middle frontal gyrus. With this direct multivariate test for changes in patterns of activity, we replicate and extend earlier findings of reduced prefrontal lateralisation in ageing. These findings extend conclusions based on conventional analyses, and support the notion that ageing alters the spatial deployment of neuronal activity, to render it less spatially coherent and regionally specific. This greater distribution of activity in older adults was also linked to poorer individual memory performance, suggesting that it reflects neural ageing, rather than adaptive compensatory responses.

## Graphical abstract

**Figure:**
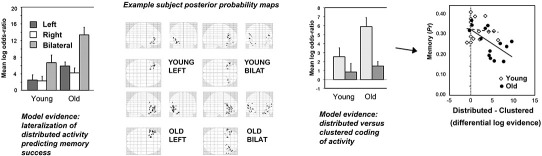

**Highlights:**

► A Bayesian decoding analysis explored functional reorganization in PFC in aging. ► Multivariate models were compared of patterns of episodic encoding-related activity. ► In older adults activity was more distributed spatially and less lateralized. ► Distributed coding in older adults was associated with poorer memory. ► This direct inference defines different patterns of activity according to age.

## Introduction

Ageing typically brings with it a decline in cognitive function, with a notable impairment in memory for the details of specific events (episodic memory; EM) (Tulving, 1983, Yonelinas, 2002). A major research challenge is to understand this decline and the prominent individual differences that characterise it, raising the possibility that there are specific neural mechanisms and resources that support good function in ageing. Before the advent of functional neuroimaging, episodic memory decline in ageing was assumed to be due simply to a loss of function in critical regions and networks, particularly in prefrontal cortex (PFC) and medial temporal lobes (MTL). Indeed, as this lesion model predicts, older adults can show ‘under-recruitment’, i.e., activity reductions relative to the young. Strikingly, however, they also frequently activate additional regions or networks (‘over-recruitment’ or ‘over-activation’; Cabeza, 2002, Grady and Haxby, 1995, Park et al., 2001, Reuter-Lorenz, 2002). In episodic memory, this may take the form of bilateral rather than unilateral activity in prefrontal cortex (Cabeza, 2002, Cabeza et al., 1997, Duverne et al., 2009, Grady et al., 1994; Logan et al., 2002, Madden et al., 1999, Morcom et al., 2003, Reuter-Lorenz et al., 2000). In memory and in visual object recognition, older adults may also show more distributed activity within a given region (Carp et al., 2010a, Carp et al., 2010b, Duverne et al., 2008, Morcom et al., 2007, Park et al., 2004, Voss et al., 2008). Findings such as these mean that cognitive decline in older adults cannot be attributed straightforwardly to an impairment in networks specialised for EM and other functions in the young (Anderson et al., 2000, Cabeza et al., 1997, Grady et al., 1994; Reuter-Lorenz et al., 2000, Madden et al., 1999).

This apparent functional reorganisation has generated great interest, because it may reflect adaptive, compensatory changes that support successful cognitive ageing (Grady and Haxby, 1995). Current debate focuses on whether reorganisation reflects these beneficial changes, or whether it directly reflects age-related neural changes that are deleterious to cognitive function (e.g. Cabeza et al., 2002, Duverne et al., 2009, Gutchess et al., 2005, Logan et al., 2002, Park and Reuter-Lorenz, 2009, Reuter-Lorenz and Lustig, 2005). Based on imaging findings, alternative theoretical accounts describe possible patterns of reorganisation in terms of differential activity in multiple regions according to age. For example, adaptive PFC responses may compensate for age-related changes in MTL (Dennis et al., 2008, Gutchess et al., 2005, Park et al., 2003), or in posterior cortex (Carp et al., 2010a, Davis et al., 2008, Payer et al., 2006). Other accounts describe a reduction in the specificity of activity patterns across hemispheres (Logan et al., 2002) or more generally (Carp et al., 2010b).

Resolving the debate about the causes and behavioural impact of functional reorganisation depends on establishing how the patterns of activity vary with age under different experimental conditions. This requires pattern level inferences; about the relative location and distribution of activity. However, such inferences are not well supported by current univariate or multivariate analyses, which do not test hypotheses about the distribution of activations across regions. In order to bridge this gap, we employed a recently developed analytic method that permits Bayesian model comparison across alternative multivariate patterns of activity that may differentially predict a psychological variable (Friston et al., 2008). Using this multivariate ‘decoding’ approach, and a rich prior dataset for which univariate age-related differences have already been well characterised (Morcom et al., 2003), we were able to ask, and answer, the novel question: which spatial activity patterns best predict successful memory formation? This enabled us to determine directly whether activity is more distributed with ageing, and whether it is less lateralised.

A characteristic of neuroimaging data that (until recently) has been under-exploited is the large number of observations distributed across the brain; the very observations that have led to the notion of over-recruitment. Mass-univariate analyses identify individual voxels in which the magnitude of task-related activity differs according to age and so support inferences about changes in functionally specialised regions, irrespective of changes elsewhere (Friston et al., 1991). Inferences based on fMRI about neural responses in multiple regions are confounded by the fact that regions may differ in their neurovascular coupling (Henson, 2005, Miezin et al., 2000). A useful and important exception has been the use of laterality analyses, directly comparing activations from homologous left- and right-sided regions, whose hemodynamic characteristics can be assumed to be similar (Gur and Chin, 1999). These have supplemented informal observations, providing evidence for age-related differences in functional lateralisation (Reuter-Lorenz et al., 2000). However, in order to test hypotheses about *patterns* of activity a multivariate approach is needed (e.g., McIntosh et al., 1996, Worsley et al., 1997). With methods such as partial least squares, task-specific networks can be identified in young adults, and shown to differ with age (e.g. Cabeza et al., 1997, Grady et al., 1999, McIntosh et al., 1996, Vallesi et al., 2010). In the domain of episodic memory, such studies have revealed that older adults recruit certain networks to a greater extent than the young, suggesting that over-recruitment is associated with changes in large-scale brain systems. These analyses demonstrate differences in the responses of individual networks to a psychological variable, thus revealing changes in functional integration.

These complementary approaches have generated a number of important hypotheses about age-related functional reorganisation. These hypotheses are not mutually exclusive, but predict distinct changes in the patterns of activity across multiple regions and networks. Thus, they depend on pattern level inference, asking questions about the nature of the patterns themselves. Such questions are difficult to address using existing methods that ask whether a psychological variable differentially predicts activity in a region or network according to age. We wanted to ask whether the pattern of activity changes with age and, critically, *how* it might have changed. These questions pose a different kind of problem. In principle, they rest on model comparison and selection. Such approaches have already been implemented in neuroimaging, both for classical (*F*-tests) and for Bayesian inference, for example in the comparison of different dynamic causal models (see Penny et al., 2006). However, these models differ in terms of how cognitive processing predicts brain responses in one or more regions, not in terms of how the distribution of regional brain responses predicts cognitive processing.

In the case of age-related over-recruitment, we want to infer which patterns predict, for example, mnemonic processing. In other words, our hypothesis or model maps from patterns of physiological activity to cognitive quantities. Such problems require ‘decoding models’ rather than ‘encoding models’. Using decoding models, one can ask which patterns predict a psychological variable. In the case of perception, decoding models consider how brain activity (Y) represents or *causes percepts*, for which stimulus presentation (X) acts as a surrogate (i.e., Y → X); as opposed to the standard encoding model, in which stimuli *cause brain activity* (i.e., X → Y). In the present context, a decoding model asks the natural question: what patterns of brain activity predict successful later memory? Decoding analyses are now used widely in neuroimaging when the goal is to classify or ‘read’ psychological states from imaging data (Haynes and Rees, 2006, Norman et al., 2006). Recent developments in analytic methods allow the identification of such models, using procedures developed for other ill-posed inverse problems such as EEG source localisation (Friston et al., 2008). Using parametric empirical Bayes, competing hypotheses about the mapping between patterns of activity and a psychological variable can be addressed using Bayesian model selection. Here, we apply these multivariate Bayesian (MVB) methods to the problem of over-recruitment in ageing. This enables a formal characterisation of patterns of cortical coding, which may address core questions about neurocognitive ageing. Using MVB, we investigated functional segregation and lateralisation within PFC, during episodic memory formation.

**Fig. 1:**
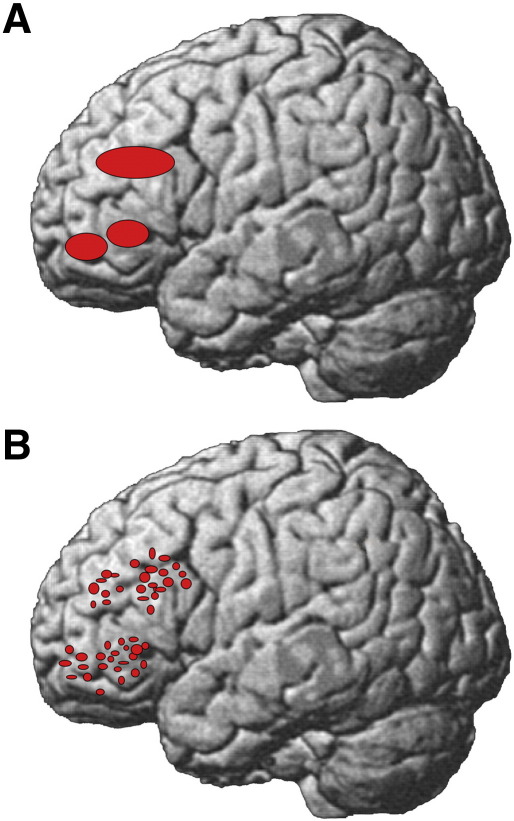
Spatial models of cortical coding. A. Clustered representations: neurons that respond in the same way are clustered together spatially, so a macroscopic region can be identified with functional selectivity. B. Distributed representations: neurons that respond in the same way are more distributed spatially, so a macroscopic region may contain neurons that have different functional properties.

A reduction in functional segregation in older adults, at a scale detectable with fMRI, has been suggested by findings that activity is more distributed within a cortical region than in the young. Fig. 1 illustrates the difference between spatially distributed and spatially clustered activity. More distributed activity may also reflect a loss of functional specialisation; i.e., de-differentiation (Park et al., 2004). Alternatively, or also, changes in functional segregation might reflect differences in the spatial deployment of specialised neural responses, without any change in specialisation (see Reddy and Kanwisher, 2006). The possibility of changes reflecting de-differentiation within PFC has received very little attention until very recently. This is surprising given this region has been implicated in both age-related EM impairment and cognitive decline more generally (West, 1996); and the claims we have already noted, that over-recruitment in this region in particular may reflect compensatory changes (Grady and Haxby, 1995). Furthermore, a prominent computational account of PFC ageing links declining memory function with representational de-differentiation due to a decrease in neural signal-to-noise (Li et al., 2001, Li et al., 2005). Consistent with the presence of such changes in PFC, a recent study by Carp et al., 2010a, Carp et al., 2010b found that lateral frontal activity during passive viewing of objects in different categories was less functionally specialised in older adults, just like activity in the posterior cortical regions known to be critical for object recognition. However, it is unknown whether this activity is distributed differently within PFC, nor whether such changes are seen in tasks such as EM encoding that are known to depend on PFC.

A number of studies have reported age-related changes in functional lateralisation within PFC during episodic memory and retrieval, working memory, and in other tasks, with less lateralised activity in older than in young adults (for reviews see Cabeza, 2002, Grady and Haxby, 1995, Park et al., 2001, Park and Reuter-Lorenz, 2009, Reuter-Lorenz, 2002). Age-invariant prefrontal lateralisation has also been reported (e.g. Morcom et al., 2007); see (Obler et al., 1984, Rajah and D'Esposito, 2005). It is not yet clear how such findings reflect the processing demands of the task and characteristics of the participants (Cappell et al., 2009, Rajah and D'Esposito, 2005, Reuter-Lorenz and Cappell, 2008, Schneider-Garces et al., 2010). To examine this, it is necessary to characterise the patterns of change; for example, to test hypotheses that predict whether contralateral recruitment is seen in homologous regions or in a different prefrontal region (Rajah and D'Esposito, 2005). These hypotheses require pattern-level inference. Importantly, the way that lateralisation in ageing is assessed means lateralisation may also be sensitive to changes in functional segregation within the regions considered. Analysis of the same small regions of interest may not reflect activity in younger and older adults in the same way if the activations are distributed differently. In a previous analysis of activity during successful episodic memory formation, we demonstrated reduced lateralisation in an old relative to a young group, in three prefrontal regions, based on a conventional mass-univariate analysis (Morcom et al., 2003). In the current study, we examined this dataset to determine whether these findings generalise to wider regions of PFC, and whether they still hold, once potential differences in functional segregation are taken into account.

We used MVB to test which patterns of activity during episodic memory formation predict performance on a subsequent memory test. Our regions of interest (ROIs) were left-sided (and homotopic right-sided) PFC areas that are consistently engaged during successful EM formation in the young (Wagner et al., 1998a). Activation in these regions was age-invariant in the conventional SPM analysis of these data, the ROIs encompassing age-invariant left-sided peaks of activity (Morcom et al., 2003; Supplementary material). In the first analysis, we compared different models of the spatial pattern of activations within the left prefrontal ROIs for younger and older adults, asking whether patterns predicting episodic memory were better characterised by distributed or clustered spatial models. We predicted that activity would be more distributed in older than younger adults within the same regions of interest, consistent with altered functional segregation, and in keeping with earlier findings in inferior temporal cortex (Park et al., 2004, Voss et al., 2008) as well as in prefrontal cortex (Carp et al., 2010b). Second, using the best spatial model, we compared unilateral with bilateral models, asking which better predicted mnemonic processing. We predicted that bilateral patterns of activity would be better predictors in the older group, extending earlier findings (i.e., the predictive information in the activity of right and left homologous regions would be greater than right or left regions alone).

## Materials and methods

A summary of the participants, experimental design, behavioural procedures and data acquisition is given here. Full details of the methods, along with the results of the univariate SPM analysis, can be found in Morcom et al. (2003).

### Participants

Participants were 28 healthy community-dwelling right handed adults recruited from two age groups. There were 14 in the young group (18–29 years; mean = 21, SD = 1.6; 3 males) and 14 in the older group (63–74 years, mean = 68, SD = 3.3; 5 males), matched for education. Participants underwent a short screening battery of neuropsychological tests, which indicated a typical pattern of age-related impairment in episodic memory and fluid ability; despite a slight increase in verbal IQ as estimated by the National Adult Reading Test (Nelson, 1982).

### Memory task and procedure

The experimental procedure consisted of an incidental study task followed by recognition memory tests. Stimuli were nouns between 4 and 9 letters in length, with a frequency of 1–30 per million (Kucera and Francis, 1967). They were taken from a pool of 557 such stimuli and their allocation to study and test lists was counterbalanced across subjects. Study and test lists also included 2 initial filler items.

Scanning took place during the study task only. Subjects made living/non-living decisions about 160 single nouns. These critical items were shown individually in pseudo-random order, interspersed with 80 fixation-only trials. Words were each presented for 650 ms, preceded by a warning signal and followed by fixation. The duration of fixation trials and item trials was 3.0 s. Two study task blocks were completed with a short break with continuous scanning, each with 122 trials.

Studied words were then re-presented in a post-scan recognition test, along with 80 new words (half after a delay of approx. 10 min and half after approximately 40 min). At each test delay, there were two test blocks of 62 trials. Data presented here are combined across the two test delays. In the recognition test, words were preceded by fixation, displayed for 300 ms, and followed by a blank screen. The time between the subject's response and the next stimulus was 3.0 s. Subjects judged whether words were studied (‘old’) or not studied (‘new’), also reporting their level of confidence, using 4 manual key-press responses. Responses indicated whether each word was confidently judged to be old, non-confidently judged to be old, confidently judged to be new, or non-confidently judged to be new.

### fMRI data acquisition

For each participant, a single time series of 337 echoplanar images was acquired using a 2 T Siemens VISION system (Siemens, Erlangen, Germany). Images comprised 32 2.5 mm thick axial slices separated by 1.5 mm, with a repetition time (TR) of 2.43 s.

### Univariate SPM analysis

Pre-processing and the original data analysis were carried out using Statistical Parametric Mapping (SPM99, www.fil.ion.ucl.ac.uk/spm/software/spm99/; see Morcom et al., 2003). The fMRI data were spatially realigned and slice-time corrected. They were then normalised to the SPM EPI template using nonlinear basis functions (Ashburner and Friston, 1999), and smoothed with a 10 mm full width half maximum (FWHM) isotropic Gaussian kernel.

The MVB analysis required that each participant's first-level general linear model (GLM) and SM contrasts be re-computed using a more recent version of SPM; SPM5 was used for this, whilst the final MVB analyses were computed using SPM8 (www.fil.ion.ucl.ac.uk/spm/software/spm8/; release r3683). These GLMs comprised covariates representing the onsets of each event type, convolved with a canonical hemodynamic response function (HRF) and a delayed HRF shifted by 2.43 s. Covariates for the delayed HRF were orthogonalised with respect to those for the canonical HRF so that shared variance was attributed to the canonical HRF (see Morcom et al., 2003). The results were broadly unchanged relative to the earlier published results of the SPM99 analysis (see Morcom et al., 2003 and Supplementary material).

Parameter estimates for each condition and covariate were calculated from the least squares fit of the model to the data, following pre-whitening based on an enhanced noise model (AR(1) + white noise; Friston et al., 2002). Each session was high-pass filtered to 1/128 Hz and proportionally scaled to a global mean of 100. We included confounding event types and six movement-related covariates generated during realignment. Linear contrasts of parameter estimates for SM effects were then computed, comparing activity for words later remembered confidently with those forgotten (i.e., not confidently remembered). This definition of ‘remembered’ was the same as in the previous conventional analysis of this dataset, as well as other subsequent memory studies (e.g. Duverne et al., 2009, Morcom et al., 2003, Otten et al., 2001, Wagner et al., 1998b). It minimises the contamination of the analysis of brain activity associated with successful encoding by activity elicited by items later recognised on the basis of ‘lucky guesses’. In the present study, non-confident discrimination of old from new items was only slightly (though significantly) better than chance, with no interaction with age (mean 0.11 hits and 0.8 false alarms; see Morcom et al., 2003).

### Regions of interest

Anatomical ROIs were specified in lateral prefrontal cortical regions from the AAL template, using WFU PickAtlas. This identifies the entire named region based on the Talairach Daemon database, extended and corrected for MNI space (Maldjian et al., 2003, Tzourio-Mazoyer et al., 2002). These included: Anterior Inferior Frontal Gyrus (aIFG; pars triangularis/orbitalis), posterior Inferior Frontal Gyrus (pIFG; pars opercularis) and Middle Frontal Gyrus (MFG). The ROIs were selected to characterise the regions of theoretical interest, without being biassed with respect to the magnitude of univariate SM effects according to age group. For the purposes of initial model selection, left ROIs were used (see MVB analysis). Unlike the right lateral PFC, these regions encompassed key age-invariant SM peaks in the conventional SPM analysis on the left (the right PFC ROIs encompassed their homologues on the right; (Morcom et al., 2003); Supplementary material, Fig. 2). A laterality analysis had shown that the magnitude of SM effects in the older age group was greater in the right ROIs, relative to those on the left (Morcom et al., 2003).

**Fig. 2:**
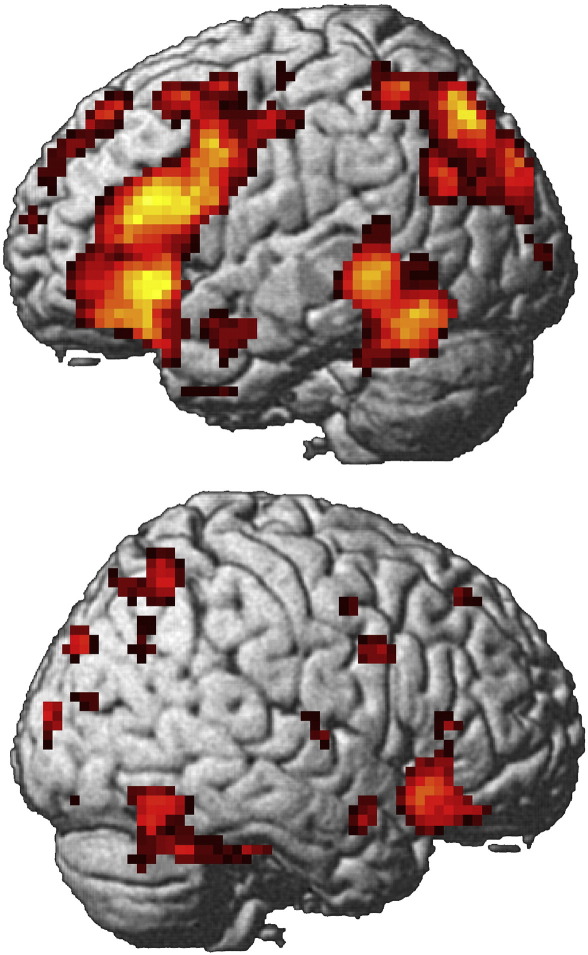
Age-invariant subsequent memory effects from conventional SPM5 analysis, rendered onto the MNI reference brain. The positive main effect of SM across both age groups was exclusively masked with the bidirectional interaction of SM × age group at *P* < 0.05 uncorrected, and the resultant SPM was thresholded at *P* < .001, cluster size > 5 for comparability with original results of Morcom et al. (2003).

### MVB analysis

We then fitted a set of MVB decoding models for each participant to test hypotheses about the spatial distribution of SM effects. Each MVB model maps many (physiological) data features to a (psychological) target variable – here, subsequent memory – using a simple hierarchal or parametric empirical Bayes model (PEB; see Friston et al., 2008). Empirical priors on the data features (voxel-wise activity) are formulated in terms of patterns over the features. Each MVB model is based on the same design matrix (X) of experimental variables used for the conventional SPM analysis (Morcom et al., 2003) and data (Y). However, the experimental variables furnish the target variable and the imaging data provide the predictor variables. The design matrix is partitioned into the contrast to be decoded and its null space (i.e., confounds), and the target and predictor variables then projected onto the null space of the confounds to give variables with confounds removed.

Because decoding models that operate on large number of voxels (relative to scans) are ill-posed, MVB uses the priors on patterns of voxel weights (that map from each voxel to the target variable) as constraints in a second level of the hierarchical model. The pattern weights are treated as second-level random effects, inducing empirical priors over the voxel weights. These priors are specified as a prior spatial covariance, which is factorised in two components: the patterns U, and the variances of the pattern weights. The nature of the patterns determines the nature of the model, allowing different patterns (models) to be compared in terms of their Bayesian model evidence.

This model embodies an overall sparsity (hyper) prior in pattern space: individual patterns are expected to contribute sparsely to the decoding, so that a few make a large contribution, whilst most make a small contribution. The pattern weights (unknown parameters of the mapping of Y to X) are treated as a nested set, in which each subset has the same variance. By construction, the variance of a pattern weight in a subset is always greater than that of a pattern weight in its superset (which contains more patterns). This nested partition of patterns weights is optimised using a greedy search and a standard variational scheme under the Laplace assumption (Friston et al., 2007). The greedy search starts with a classical minimum norm solution (where all pattern weights have the same variance) and iterates the scheme so that successive bipartitions isolate the subset with the largest pattern weights. The free energy of successive partitions increases until the optimum set size is reached; at which point the free energy provides an upper bound on the log-evidence. The set of patterns chosen constitutes a model or hypothesis about the nature of the mapping between voxels (in Y) and the target variable (in X). In this way, MVB decodes the neuronal representation or cause of the psychological target variable according to each model of data features. The evidence for different models can then be compared (see Friston et al., 2008).

In this work, we tested distributed and clustered spatial models. In a distributed model, each pattern is an individual voxel, so partitions are subsets of individual voxels. In a clustered spatial model, each pattern is a cluster of voxels with smooth local support (defined by a Gaussian with FWHM = 4 mm^3^), so partitions are subsets of smooth contiguous clusters. For inference at the group level, we treated participant-specific log-evidences for each model as summary statistics for classical inference using analysis of variance (ANOVA with alpha = 0.05, and Greenhouse–Geisser corrections for non-sphericity where appropriate; (Greenhouse and Geisser, 1959)). The dependent measure was the difference in the free energy bound on the log-evidence for the model and a null model — one in which there are no patterns and no mapping. The null model will be the best model (with a minimum complexity cost) if regional activity is uninformative in predicting the psychological variable. This difference in log-evidence is the log (marginal) likelihood ratio test or log Bayes factor for comparing the models.

Our model selection and comparison proceeded in two stages: first, different models of the spatial deployment of activity within each left PFC ROI were compared within and across age groups. Second, using the best spatial model from the first stage, unilateral models were compared with bilateral models encompassing homotopic left and right PFC ROIs. In other words, the models were defined by patterns within left, right or both ROIs. The purpose of our analysis was to compare different models of the same memory performance based on their Bayesian model evidence. However, we can also quantify the performance of these models in an intuitive way by evaluating the proportion of variance in memory performance that can be explained by distributed haemodynamic responses. This is simply the R-squared statistic based upon the conditional estimates of any model. In aIFG, the average proportion of variance explained under the clustered unilateral models was 19%/19% (for young/older groups), under distributed unilateral models it was 23%/29%, and under distributed bilateral models it was 35%/42%. In DLPFC the corresponding values were 20%/17%, 28%/28% and 44%/48%. This measure of model performance corresponds to the classification or regression accuracy; usually accessed using cross validation schemes. However, cross validation is unnecessary in our analysis due to the parametric assumptions: these mean that we can quantify decoding accuracy directly using the R-squared statistic. Importantly, the differences in decoding accuracy do not tell us about the quality or relative evidence for different models. This is because, unlike model evidence, accuracy measures do not reflect differences in the complexity of models.

At both stages of model comparison, where MVB comparisons gave rise to significant between-group differences, we investigated their relationship to behaviour and to successful ageing, by assessing whether patterns of brain activity predicted individual differences in cognitive test performance. For each MVB analysis, differences in the log-evidence between models were entered as predictors in simple linear regression analyses for each of four behavioural dependent variables. t-Tests on regression slopes or their differences were computed across and then between age groups (Bonferroni-corrected alpha level = 0.00625 for 8 tests). The behavioural variables were selected from summary measures of performance on the experimental memory task, and from a neuropsychological battery completed by participants in a separate test session: episodic memory task measures were an index of discrimination of studied from unstudied items (*Pr*; Snodgrass and Corwin, 1988) and a measure of response time (RT). To obtain an RT measure that reflected general processing speed, known to decrease with age (Salthouse, 1996), as opposed to their speed of memory retrieval, an average was taken of RT for all conditions in the subsequent memory test, for both correct and incorrect responses, for which observations were available for all participants (i.e., confident hits and correct rejections, and misses and false alarms). Neuropsychological measures assessed crystallised and fluid IQ using the National Adult Reading Test Full Scale IQ estimate (Nelson, 1982), and a short version of the Raven's Advanced Progressive Matrices (Raven et al., 1994, Spearman, 1927; see Morcom et al., 2003 for further details).

## Results

### Comparison of distributed and clustered spatial models

**Fig. 3:**
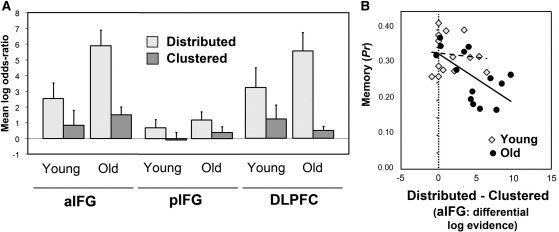
Spatial coding of SM effects in left PFC. A. Bayesian model comparison. Plots show mean log-odds ratio (difference in log-model evidence for each model vs. the null model) by age group, for the clustered and distributed spatial models. Error bars represent the standard error of the within-group mean. B. Brain–behaviour analysis for aIFG. The x-axis shows mean log-odds ratio for distributed vs. clustered models, and the y-axis shows memory performance in terms of *Pr* (pHits–pFalse alarms).

Fig. 3 summarises the log-evidence for the competing spatial decoding models in the three left prefrontal ROIs, for the young and older age groups. ANOVA with factors of group (young/old), region (aIFG/pIFG/DLPFC) and model (distributed/clustered) revealed main effects of region (*F*(1,26) = 45.3, *P* < .001) and model (*F*(1.5,39.6) =15.5, *P* < .001) and an interaction of region × model (*F*(1.9,48.7) =18.0, *P* < .001). These significant between subjects results reflected a greater evidence for distributed over clustered models in general, and for aIFG and DLPFC coding over pIFG models in general. The interaction suggested that the advantage of distributed models was more marked in aIFG and DLPFC. A 2-way interaction of group × model (*F*(1,26) = 6.9, *P* < .05) also reflected the greater advantage of distributed over clustered models in the older age group. However, all of these effects were modified by a 3-way interaction (*F*(1.9,48.7) = 5.7, *P* < .01); the age-related difference in the log-evidence for distributed compared to clustered models varied according to region, being reliable and of similar magnitude in aIFG and DLPFC, and not reliable in pIFG.

Follow-up (post hoc) analyses explored these effects region by region. In left aIFG, ANOVA with factors of group (young/old) and model (distributed/clustered) showed a main effect of model (*F*(1,26) = 35.4, *P* < .001) and an interaction of model with group (*F*(1,26) = 6.8, *P* < .05). This shows that model evidence was greater for distributed than for clustered models, but this difference varied with age. t-Tests confirmed that the evidence for distributed models was greater in both age groups (*T*(13) = 2.8, *P* < .05 for young; *T*(13) = 5.3, *P* < .001 for old). The group-wise difference in log-evidence was also significant for distributed but not for clustered models (*T*(26) = 2.1, *P* < .05; *T* < 1, respectively). Findings in the left DLPFC were very similar. ANOVA again revealed a main effect of model (*F*(1,26) = 36.5, *P* < .001) and an interaction of model with group (*F*(1,26) = 6.8, *P* < .05). Again, follow-up tests by age group confirmed that evidence was greater for distributed than clustered models for both age groups (*T*(13) = 2.8, *P* < .05 in young; *T*(13) = 5.3, *P* < .001 in old), although tests by spatial model did not reveal separately reliable group differences. In pIFG, however, ANOVA showed only a main effect of model (*F*(1,26) = 8.8, *P* < .001; *F* < 1 for other effects), the evidence being greater for distributed compared with clustered models.

In summary, there was greater evidence for distributed relative to clustered spatial models in all three ROIs, but, importantly, the advantage for distributed over clustered models was significantly greater in the older age group in aIFG and DLPFC (Fig. 3). Because evidence for distributed models was greater than clustered models in both age groups, these models were selected for the laterality analysis.

Two separate brain–behaviour analyses used the log-evidence for distributed (relative to clustered) models in aIFG and in DLPFC as the predictor variables. There were no reliable findings for DLPFC, but there were for aIFG. These results are summarised in Fig. 3. Across the two age groups, relative log-evidence for distributed coding (models) reliably predicted both low *Pr* – poorer memory – and slower RTs (beta = −.54, *T(*26) = 3.30, *P* < .005; beta = 0.54, *T*(26) =3.28, P < .005). Group-wise analysis revealed that the relationship with *Pr*, but not RT, differed reliably between young and older groups (for *Pr*, regression slope = − 0.10 and −.61, respectively; *T*(24) =16.04, P < .001; for RT, regression slope = .29 and .65, respectively; *T*(24) = − 0.57, n.s.). A follow-up (uncorrected) test indicated that the relationship for *Pr* was only separately reliable in the older group (*T*(12) = 2.68, P < .05). Further follow-up tests also examined associations between Pr (in the older group) and RT (in both groups combined) and the model evidences for clustered and distributed models separately. These confirmed that both of these brain–behaviour associations reflected greater evidence for distributed over clustered models in poorer performers (for Pr in older group, beta = −.53, *T*(13) = − 2.19, *P* < .05; for RT in groups combined, beta = .47, *T*(26) = 2.68, *P* < .05), as opposed to relatively greater evidence for clustered models in better performers (all *T* < 1). Regression analyses for NART IQ and for Raven's scores did not reveal significant relationships.

### Comparison of unilateral and bilateral distributed models

**Fig. 4:**
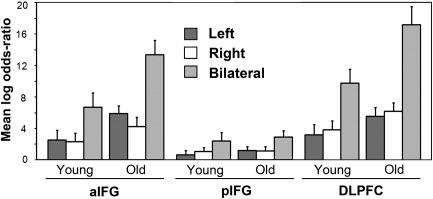
Lateralisation of SM effects in PFC for distributed spatial models: Bayesian model comparison. Plots show the mean log-odds ratio by age group, for left, right and bilateral models (see Fig. 3 for details of measures).

**Fig. 5:**
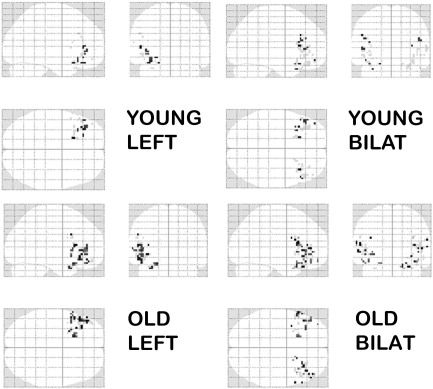
Laterality of coding of SM effects in left aIFG: maximum intensity projections (MIPs). MIPs show posterior probability maps based on voxel weights for the distributed models by ROI laterality in example old and young subjects. The voxel weights are the voxel-wise fitted decoding parameters that characterise the mappings between each voxel and the target variable according to the model (see MVB analysis and Friston et al., 2008).

Fig. 4 summarises laterality effects on the log-evidence for distributed models, according to age group, in the three prefrontal ROIs. Fig. 5 illustrates the patterns of activity for left and bilateral distributed models in aIFG for two example young and older adults. The laterality effects were quantified using ANOVA with factors of group (young/old), region (aIFG, pIFG, DLPFC) and laterality (L/R/bilateral). This revealed effects of laterality; a main effect (*F*(1.9, 48.9) = 65.1, *P* < .001), 2-way interactions with group (*F*(1.9, 48.9) = 5.2, *P* < .05) and region (*F*(3.2,82.1) = 21.8, *P* < .001), and a 3-way interaction (*F*(3.2,82.1) = 2.9, *P* < .05). Evidence was greater for bilateral than for unilateral models — both L and R, and this difference was more pronounced in the older group and in aIFG and DLPFC. Greater evidence for a contribution of these regions to SM in the older group was also reflected in main effects of group (*F*(1,26) = 4.6, *P* < .05) and region (*F*(2.0,51.4) = 32.6, *P* < .001), and an interaction of region × group (*F*(2.0,51.4) = 3.7, *P* < .05). Model evidence was greater in the older group in aIFG (*F*(1,26) = 5.1, *P* < .05) and DLPFC (*F*(1,26) = 4.6, *P* < .05), these regions differing from pIFG in this respect (*F* < 1).

Post hoc ANOVAs by region indicated that the age-related difference in laterality was reliable only in the latter two regions (*F*(1.9,50.1) =4.4 and *F*(1.9,50.1) = 6.3, respectively, *P* < .05; for pIFG, *F* < 1). Within aIFG, pairwise comparisons showed reliable differences between left and bilateral models in both age groups (*T*(13) = 5.1, *P* < .001 and *T*(13) =4.6, *P* < .001), but the difference in log-evidence was greater in the old group (see Fig. 4). Likewise, right and bilateral models differed for both age groups (*T*(13) = 4.3, *P* < .005 and *T*(13) = 5.6, *P* < .001). There was a similar picture in DLPFC (for left vs. bilateral, *T*(13) = 5.7, *P* < .001 and *T*(13) = 7.5, *P* < .001; for right vs. bilateral, *T*(13) = 5.8, *P* < .001 and *T*(13) = 6.2, *P* < .001). In pIFG, evidence was greater for bilateral than unilateral models across both age groups but the groups did not differ in laterality (for L vs. bilateral, *T*(27) = 3.9, *P* < .001; for R vs. bilateral, *T*(27) = 3.8, *P* < .001).

Brain–behaviour analyses used the log-evidence for bilateral coding in aIFG (relative to left unilateral) as the predictor variable. However, regression analyses did not reveal any reliable relationships between this and the four behavioural measures, either across the two groups or in terms of age-related differences.

## Discussion

We provide direct evidence that the patterns of prefrontal activity associated with successful episodic memory formation change with age. In anterior IFG and dorsolateral PFC, these activity patterns were more distributed (fragmented) in older than in younger adults, and more bilateral. The findings support the notion that ageing alters the spatial distribution of neuronal activity, to render it less spatially coherent. This result extends earlier observations on visual object recognition and on memory. Importantly, our analysis permits the inference that the spatial patterns of activity differ across the regions considered, and supports formal inter-regional comparisons. The increased evidence for bilateral coding, demonstrated in the older group, replicates and extends results from previous conventional analyses of this and other data. It demonstrates age-related de-lateralisation, under distributed coding within PFC. This illustrates how the comparison of multivariate models of regional coding can address hypotheses about functional segregation and reorganisation in ageing.

### Distributed or clustered coding and functional segregation

Our first aim was to examine group differences in functional segregation; the anatomical arrangement of neuronal populations according to their functional properties (Zeki, 1990). The comparison of different spatial models within lateral PFC revealed greater evidence for distributed relative to clustered coding in the older adults in both aIFG and DLPFC, but not in pIFG. The models embodied alternative spatial priors, characterised as sets of distributed or clustered patterns. Distributed patterns were single voxels, whilst clustered patterns comprised smooth mixtures of contiguous voxels. Greater evidence for distributed than clustered models implies that activity in a set of single voxels predicts subsequent memory effects better than the activity of smooth clusters of neighbouring voxels. The greater evidence for distributed models in the older group therefore indicates a greater difference between neighbouring voxels (heterogeneity) in this respect.

The MVB analysis does not examine brain activity in the same way as a conventional mass-univariate analysis. In keeping with the different questions it addresses, the spatial patterns of activity revealed need to be interpreted differently. As noted earlier, mass-univariate SPM analysis of SM effects in these PFC regions revealed extended clusters of activity in both age groups with responses of equivalent magnitude and a spatially smooth appearance (Morcom et al., 2003); Supplementary material). Why might MVB reveal multiple smaller foci that are not apparent in mass-univariate results? How does this relate to prior observations of a greater number of supra-threshold voxels in a region, or in a cluster, in older than in young adults? As has been pointed out in the context of data-driven multivariate decoding analysis, multiple regions not individually associated with a behavioural variable may jointly predict that variable (see Haynes and Rees, 2006, Kriegeskorte and Bandettini, 2006). With its sparsity priors in pattern space, MVB will select the smallest subsets of patterns – here voxels or smooth clusters – that have the greatest variance; i.e. those few that jointly best predict the behavioural variable. Conversely, in mass-univariate analysis model estimation is independent over voxels, and there is no penalty for including multiple voxels encoding the same information. In addition, the use of minimum cluster size thresholds (> 5 in Morcom et al., 2003) increases the likelihood of clustering of significant voxels. Thus, mass-univariate analyses cannot detect differences in multi-voxel coding, and may not reveal differences in the spatial distribution of activity. Such approaches have in some cases revealed a greater number of supra-threshold voxels within a region in older adults (e.g., Solbakk et al., 2008, Altered prefrontal function with aging: insights into age-associated performance decline). These results show local over-recruitment at a given threshold, and suggest that the spatial distribution of activity may differ, but do not actually provide information about spatial distribution. Our approach can formally demonstrate differences in the spatial distribution of activity and, uniquely, supports direct inferences about spatial differences in coding.

### Functional segregation and specialisation in ageing

Our comparison of clustered and distributed models suggests that patterns of activity predictive of later successful remembering are more spatially distributed in older adults than in the young. Three earlier neuroimaging studies have reported related findings in the domain of visual object recognition (Carp et al., 2010b, Park et al., 2004, Voss et al., 2008; see also Carp et al., 2010a, Payer et al., 2006). These studies primarily addressed functional specialisation; i.e., the level of specificity or distinctiveness of neural codes for different categories of information. A landmark study of object recognition responses in ventral visual cortex (VVC), by Park et al. (2004), suggested that voxels responding to faces, houses and other categories overlapped to a greater extent in older adults (see also Voss et al., 2008). Those findings were recently extended in the same dataset, using MVPA and a cross-map correlation measure of distinctiveness, which suggested that activity in PFC as well as in VVC carried less category-specific information in the older age group, in keeping with age-related functional de-differentiation (Carp et al., 2010b; see also Carp et al., 2010a). One of Park et al.'s (2004) findings also speaks to the issue of functional segregation: in addition to age-related reductions in the category-specificity of individual voxel responses, there was greater intermixing within VVC of voxels sensitive to different categories, for example more face-sensitive voxels within primarily scene-sensitive regions. This is suggestive of links between age-related changes in functional specialisation and segregation but does not demonstrate them directly. An important future development of our MVB approach will be formally to assess the relationship between the distinctiveness of neural representations and their patterns of spatial distribution.

The present study suggests a finer-scale organisation in the older group in prefrontal cortex; i.e. activity in individual rather than small groups of voxels. This is consistent with changes in functional segregation, where neurons in neighbouring voxels may code for different information. What kind of neural changes might give rise to this observation at the relatively large scale of several millimetres? One possibility is age-related changes in fine-scale topographies, with consequent differences in larger-scale irregularities observable with fMRI. In primary sensory cortex, the fine-scale topographic organisation of neurons according to their response preferences is well established; for example, visual orientation selectivity (Hubel and Wiesel, 1962, Hubel and Wiesel, 1968). It is thought that fMRI reveals minor irregularities in their organisation, enabling the detection of high resolution topographies as larger-scale spatial patterns of segregation at the level of voxels through biassed low-resolution sampling (Haynes and Rees, 2006, Kamitani and Tong, 2005, Op de Beeck et al., 2008). These fine-scale functional topographies are not restricted to sensory cortices; they have also been observed in higher and association cortices, including prefrontal areas (Dahl et al., 2009, Op de Beeck et al., 2008, Roe, 2009, Tanaka, 2003, Tsunoda et al., 2001).

If altered fine-scale cortical architecture in older adults is responsible for the changes in functional segregation that we observe at the voxel-scale, what impact might this have on function? Relatively little is known about the role of the brain's topographic organisation in adult cognition, although it is thought to be critical for establishing functional specialisation and connectivity during development (e.g. Bamford et al., 2010, Miller et al., 1989, Willshaw, 2006, Willshaw and von der Malsburg, 1976; but see Horton and Adams, 2005). Such organisation is ubiquitous throughout the brain, and appears to be actively maintained during adulthood through experience-dependent plasticity and synaptic remodelling (Bamford et al., 2010, Trachtenberg et al., 2002). Sensory and other cortical maps may support wiring efficiency (Chklovskii and Koulakov, 2004) and play a role in multimodal integration (Holmes and Spence, 2005). Thus, they may reflect fundamental principles of ongoing functional specialisation that may, as we have already noted, show changes in ageing. A specific possibility is that the low-level spatial organisation of cortical responses impacts on function through emergent properties of spatially-correlated and overlapping feature maps: this has been proposed as the basis for regional specialisation such as category-selectivity within VVC (Op de Beeck et al., 2008), which appears to be disrupted in ageing (Carp et al., 2010a, Carp et al., 2010b, Goh et al., 2010, Park et al., 2004). A prominent theory of PFC function also highlights the flexible and distributed nature of its neural coding, and the importance of differential coding of task-related stimulus preferences by cell assemblies within PFC regions (Duncan, 2001, Kusunoki et al., 2010). Currently, little is known about how ageing may affect these mechanisms, but recent data in rhesus monkeys have linked age-related changes in dorsolateral prefrontal macrocolumnar architecture with impaired cognitive function (Cruz et al., 2009). Thus, the differences we observe at the voxel level between spatial patterns of encoding-related activity in older and younger adults may reflect important underlying neural changes associated with functional de-differentiation and processing efficiency.

### Distributed and clustered coding: noise considerations

Given the indirect nature of the BOLD fMRI signal, it is important also to consider what impact age-related changes in either brain structure or hemodynamic properties might have on the spatial clustering of activity in older and younger adults. We cannot rule out a contribution to the present findings of changes in physiological noise or of cortical atrophy (see Samanez-Larkin and D'Esposito, 2008). Such changes might also be functionally relevant and related to the level of performance on a task; although we note that prediction of the behavioural variable by models of cortical activity was generally better, not poorer, in the older group. Extension of our approach to examine functional specialisation and de-differentiation will avoid this issue as, like the lateralisation analysis we report, comparisons of response specificity within regions will be based on the best spatial models in different age groups. In this respect, MVB has an advantage over any analytic approach in which the spatial spread of activity contributes to inference about age-related differences.

A recent study has reported a link between ‘noise’ aspects of the BOLD signal and individual cognitive differences in ageing (Sokunbi et al., 2011). Whether these reflect neural or other associated changes is a key empirical question, but as we have noted, modelling work has linked de-differentiation of neural representations in ageing with decreases in neural signal-to-noise (Li et al., 2001). It is possible that the spatial characteristics of neural fluctuations also change with age, becoming less smooth; particularly if fine-scale architecture is disrupted. Distinct from findings of either over- or under-recruitment in older adults at the level of clusters of activity, several studies investigating age-related changes in the properties of the BOLD signal have also revealed fewer suprathreshold voxels within a region in older age groups, in some cases accompanied by similar BOLD signal amplitude at peak voxels (see Samanez-Larkin and D'Esposito, 2008 for review). Such findings can reflect the presence of a greater number of voxels in a region that are noisy or exhibit negative effects relative to those at the peak voxel (Aizenstein et al., 2004, D'Esposito et al., 1999, Huettel et al., 2001). Importantly, however, ‘noise’ in this context refers to any activity uncorrelated with the behavioural variable of interest. Therefore, such observations are also consistent with changes in functional segregation and specialisation; for example, Park et al.'s (2004) finding that in older adults more voxels responded to object categories other than those they were primarily selective for.

### Lateralisation

Our second aim was to assess group differences in functional lateralisation within lateral PFC, based on the preferred spatial models from the first analysis. This comparison revealed a greater advantage in the older group for bilateral over unilateral distributed models; again the groups differed in aIFG and DLPFC, but not in pIFG. This extends findings from voxel-based laterality analyses, and from informal comparisons, that functional lateralisation within PFC is altered in ageing (Cabeza, 2002, Duverne et al., 2009, Grady and Haxby, 1995, Morcom et al., 2003, Park et al., 2001, Park and Reuter-Lorenz, 2009, Reuter-Lorenz, 2002). With the MVB analysis, we demonstrate directly that the patterns of activity predicting SM are more bilateral in older adults across these two broad ROIs, and show that these regions differ from pIFG in this respect.

As discussed in relation to the comparison of distributed and clustered models, the inferences to be made from these results are complementary to those supported by conventional analyses. Greater evidence for bilateral than unilateral models implies that within bilateral PFC there is a set of voxels that together predicts the behavioural variable better than sets of voxels within only left, or right, PFC. However, although we found evidence of greater bilaterality in the older group, we did not find reliable evidence of left-lateralisation in either group: bilateral models predicted SM better than unilateral models did in both age groups, but left and right unilateral models did not differ. This is in contrast with paired-voxel laterality analyses previously conducted in this dataset (Morcom et al., 2003). The paired-voxel analyses compared the magnitude of responses in left and right homotopic voxels within these PFC regions (because of the applied spatial smoothing, each represented a weighted average of activity within a locally extended cluster). These revealed greater left than right sided SM effects in young adults, but reduced lateralisation in the older group, with an increase in the magnitude of right-sided responses (see Duverne et al., 2009 for replication and extension of this finding).

This difference in findings likely reflects the differences between the measures used. The model evidence summary statistic we employ reflects how well multivariate activity predicts SM, not the magnitude of responses. It also reflects this prediction of the behavioural variable over whole lateral PFC regions, not individual voxels of interest. Thus, mass-univariate and paired-voxel laterality analyses together show that in both age groups, in some parts of the ROIs there are left-lateralised responses in local extended clusters, satisfying the original combined magnitude and extent threshold (Morcom et al., 2003). Right sided SM clusters were not seen in either age group at the threshold set, but the paired-voxel analysis indicated that in older adults, left-sided SM effects were more likely to be accompanied by some degree of right-sided response; i.e., greater bilaterality. However, as already noted, multiple regions may jointly carry information about a behavioural variable when their individual responses do not reliably predict that variable (Haynes and Rees, 2006, Kriegeskorte et al., 2006). Consistent with this, our multivariate analysis of responses in this dataset revealed equivalent prediction of SM by right and left lateral PFC in the young, and better prediction by bilateral PFC models. This suggests that there is substantial distributed coding across both hemispheres, as well as some extended left (and perhaps right) sided local clusters of activation. In the older group, multivariate analysis revealed more bilateral distributed coding than in the young. Given that activity in extended local clusters was also bilateral, the findings together suggest that prefrontal neural activity predicting SM is more bilateral and distributed in older than in young adults.

As mentioned in the Introduction, numerous studies have reported reduced lateralisation of prefrontal responses in older adults in episodic memory and other tasks. For episodic memory, reduced lateralisation of PFC responses in older adults has been shown during both the encoding (Duverne et al., 2009, Logan et al., 2002, Miller et al., 2008, Morcom et al., 2003, Persson et al., 2006, Rosen et al., 2002; but see Gutchess (Gutchess et al., 2005) and the retrieval phases (Backman et al., 1997, Cabeza et al., 1997); Cabeza et al., 2002, Fernandes et al., 2006, Grady et al., 2002, Madden et al., 1999, Maguire and Frith, 2003; but see Duverne et al., 2008, Morcom et al., 2007). Our results are consistent with and extend these earlier findings. They also suggest that the hypothesis that activity is less lateralised in older adults may not fully capture age-related differences in hemispheric function. With either informal assessment of thresholded maps, or with paired-voxel or paired-region laterality analyses, the degree to which activity is ‘more bilateral’ in older adults cannot be assessed unless it is clearly lateralised in the young, and this is not always the case (e.g., Cabeza et al., 2004, Grady et al., 2005). Our approach permits such comparisons. It also goes beyond paired-laterality analyses in supporting a direct and specific pattern-level inference about the degree to which activity in variously defined bilateral compared to unilateral regions better predicts a behavioural variable.

Although we did not set out with a strong hypothesis about differences between PFC sub-regions in the degree and nature of age-related changes, our data show more bilateral and distributed prefrontal coding in older adults in more anterior IFG and in DLPFC, but not in more posterior IFG. This is in keeping with the implication of these PFC regions in the kind of flexible cognitive control and selection that is impaired in ageing (Clark and Wagner, 2003, Duncan, 2001, Rajah and D'Esposito, 2005, West, 1996). That said, the overall picture in the literature is not clear. Others have reported age-related increases in right IFG activity during episodic encoding (Persson et al., 2006) and right DLPFC activity during retrieval (Fernandes et al., 2006). However, a recent review of PFC activity in functional imaging studies of memory in older and younger adults concluded on the basis of an informal meta-analysis that the evidence best supported a de-differentiation hypothesis in ventral PFC, and a right hemi-ageing hypothesis with left-sided functional compensation in dorsal and anterior PFC (Rajah and D'Esposito, 2005). Clearly, different patterns of change may occur obtain in different regions and may also generalise across tasks to different degrees. Importantly, our data also raise the possibility that these large-scale patterns of change may also be accompanied by changes in the distribution of coding within regions.

### Analysis and interpretation of functional organisation

Functional neuroimaging studies of ageing have brought to light a bewildering array of apparent changes in the brain's functional organisation, but a consistent picture has yet to emerge as to whether reorganisation is adaptive or whether it merely reflects the underlying causes of cognitive decline. We have argued that this is, in part, because these patterns of change are difficult to characterise formally, and that formal characterisation is critical for the evaluation of hypotheses about their distribution and relation to cognitive decline. Pattern-level comparisons can support new tests of the key hypotheses about brain ageing that depend on characterisation of functional organisation across different tasks and in different networks. We can ask directly whether activity in older adults is more bilateral, as here; whether it is reduced in the right hemisphere (see Rajah and D'Esposito, 2005), or whether it undergoes a posterior to anterior shift (Davis et al., 2008). Building on such comparisons, more complex hypotheses can be tested. For example, one can ask whether a posterior to anterior shift, if seen, also involves a shift to more bilateral activity, or whether ‘bilateral’ activity in older adults reflects increased prefrontal activity in unique regions not engaged in the young, or in homologous contralateral regions (Gutchess et al., 2005; see also Cabeza et al., 2002). Using MVB, we can also address the extent to which age-related shifts in patterns of activity generalise or vary across tasks, and the extent to which they track individual differences in cognitive performance.

This study provides a proof-of-concept that this is possible, using multivariate Bayesian decoding and model comparison (Friston et al., 2008). Our novel findings regarding more distributed and bilateral coding in older adults have already been discussed. What, if any, conclusions can be drawn in relation to competing hypotheses about functional reorganisation in ageing? The main question is whether or not reorganisation is adaptive, or beneficial to cognitive function (Cabeza, 2002, Logan et al., 2002, Park et al., 2004, Rajah and D'Esposito, 2005, Reuter-Lorenz and Cappell, 2008, Reuter-Lorenz and Lustig, 2005, Stern, 2009, Zarahn et al., 2007). For activity in a region to be ‘compensatory’ it must be associated with better task performance than that which is found when the activity is not present. When activity is measured in different regions in the same subjects at the same time, as here, this cannot be determined, even if the activity is associated with successful task performance. For example, in older adults right PFC SM effects may simply co-occur with left PFC SM effects due to (for example) callosal disinhibition, or de-differentiation of representations across bilateral cortices (see Morcom et al., 2003). This interpretation persists when the observations of activity are multivariate rather than univariate.

Although the MVB analysis does not by itself indicate whether or not the changes we observe are adaptive, the results of the across-subjects brain–behaviour analysis suggest that the age-related changes in the spatial deployment of SM effects, at least, may not be. Greater evidence for distributed over clustered coding in the older group is associated with poorer individual memory function. Brain–behaviour associations were not reliable for the laterality changes, but we note that in the closely similar experiment of Duverne et al. (2009), increased bilaterality of SM effects as measured using a paired-voxel comparison was seen only in poorer performing older adults. That observation is more in keeping with a mechanism linked to cognitive deterioration than with adaptive change or successful compensation (for examples of other such findings see Miller et al., 2008, Persson et al., 2006; but see Cabeza et al., 2002, Rosen et al., 2002 for different results). Clearly, systematic studies are needed to test these hypotheses directly, and the present study shows that a useful approach may be to combine such analyses with pattern-level inference using MVB.

## Conclusions

In summary, we applied a recently developed multivariate decoding analysis in a novel approach to the problem of characterising functional reorganisation in ageing. The results demonstrate that the patterns of activity predicting subsequent memory in an episodic encoding task, within two key prefrontal regions, are more locally distributed and globally bilateral in older adults. The reduction in spatial coherence of predictive prefrontal activity in older adults was also associated with poorer memory performance. These findings are consistent with changes in functional segregation and hemispheric function in ageing. The link between the greater distribution of activity in PFC and poor memory performance in older adults implicates detrimental changes associated with neural ageing, rather than an adaptive, compensatory response. This study also illustrates how pattern-level comparison of multivariate models of regional coding can provide new and direct tests of hypotheses about over-recruitment and large-scale patterns of change in ageing. This approach will enable new tests of competing models of how detrimental and adaptive changes may interact to give rise to declining or preserved cognitive function.

## Disclosure statement

We are not aware of any actual or potential conflicts of interest regarding this work.
