# Supplementary material for: Decoding episodic memory in ageing: A Bayesian analysis of activity patterns predicting memory
Source: Neuroimage. 2012 Jan 16;59(2-5):1772–82. doi: 10.1016/j.neuroimage.2011.08.071 (PMC3236995; doi:10.1016/j.neuroimage.2011.08.071)
Supplement: Supplementary file 1 — Supplementary materials. [file mmc1.doc]

# Decoding episodic memory in aging: a Bayesian analysis of activity patterns predicting memory

# Supplementary Material

Supplementary Table 1. Regions showing significant signal increases, common to both age groups, on the canonical hemodynamic response function (HRF) for words that were subsequently confidently remembered versus forgotten. The positive main effect of SM across both age groups was exclusively masked with the bidirectional interaction of SM x age group at *P* < 0.05 uncorrected, and the resultant SPM was thresholded at *P*  < .001, cluster size > 5 for comparability with the original results of Morcom et al. (2003). Location is with respect to the system of Talairach and Tournoux (1988). *Z* values refer to the peak of the activated cluster, the size of which is indicated in brackets.

| Location  (x, y, z) | Peak *Z* | Region | Brodmann area |
| --- | --- | --- | --- |
| -30 -72 42 | 5.52 (795) | L precuneus/ superior parietal gyrus | 7 |
|  |  | Large cluster incorporating sub-peaks: |  |
| -33 -60 30 |  | L precuneus | 7 |
| -24 -90 33 |  | L cuneus | 19 |
| -45 -57 -15 | 5.35 (3555) | L fusiform gyrus | 37 |
|  |  | Large cluster incorporating sub-peaks: |  |
| -45 27 -9 |  | L inferior frontal gyrus | 47 |
| -57 -42 -3 |  | L middle temporal gyrus | 37 |
| -48 9 24 |  | L middle frontal gyrus | 9/8 |
| -27 15 51 |  | L middle frontal gyrus | 6 |
| -39 21 21 |  | L inferior frontal gyrus | 45 |
| -30 -15 -15 |  | L hippocampus |  |
| -21 -12 -18 |  | L parahippocampal gyrus | 28 |
| 42 30 -9 | 4.83 (245) | R inferior frontal gyrus | 47 |
| 39 -63 -15 | 4.42 (327) | R fusiform gyrus | 37 |
|  |  | Large cluster incorporating sub-peak: |  |
| 24 -12 -24 |  | R parahippocampal gyrus | 28/34 |
| 21 -33 30 | 4.36 (74) | R cingulate gyrus | 31 |
| 12 -75 -33 | 4.18 (301) | R cerebellum |  |
| 24 -21 48 | 4.16 (29) | R cingulate gyrus | 31 |
| -12 -51 0 | 4.04 (109) | L occipital lingual gyrus | 19 |
| 57 3 -18 | 4.00 (22) | R inferior temporal gyrus | 20 |
| 15 -51 -3 | 3.96 (78) | R occipital lingual gyrus | 19 |
| 33 -60 57 | 3.94 (82) | R superior parietal gyrus | 7 |
| 21 -93 12 | 3.81 (128) | R cuneus | 17 |
| -54 6 -18 | 3.71 (46) | L inferior temporal gyrus | 20 |
| 48 6 33 | 3.65 (27) | R middle frontal gyrus | 9/8 |
| -27 -15 60 | 3.65 (11) | L precentral gyrus | 6 |
| -18 -96 6 | 3.63 (12) | L cuneus | 17 |
| 45 -81 15 | 3.58 (8) | R middle occipital gyrus | 19 |
| 18 -84 36 | 3.57 (38) | R cuneus | 19 |
| 33 -66 36 | 3.57 (18) | R superior parietal gyrus | 7 |
| 48 -12 9 | 3.55 (42) | R insula |  |
| 27 0 -39 | 3.54 (5) | R inferior temporal gyrus | 20 |
| -12 63 15 | 3.50 (10) | L superior frontal gyrus | 10 |
| -36 3 -39 | 3.47 (7) | L middle temporal gyrus | 20 |
| 0 3 33 | 3.46 (11) | Cingulate gyrus | 24 |
| -6 12 21 | 3.36 (11) | L cingulate gyrus | 33 |
| 51 0 45 | 3.35 (6) | R precentral gyrus | 6 |
| 15 12 -6 | 3.35 (6) | R putamen |  |
| 45 -30 12 | 3.28 (5) | R superior temporal gyrus | 41 |
| -18 -75 3 | 3.25 (7) | L lingual gyrus | 18 |
